# Supplementary material for: Physiological and subjective arousal to prospective mental imagery: A mechanism for behavioral change?
Source: PLoS One. 2023 Dec 12;18(12):e0294629. doi: 10.1371/journal.pone.0294629 (PMC10715665; doi:10.1371/journal.pone.0294629)
Supplement: S1 Table — (PDF) [file pone.0294629.s001.pdf]

**S1 Table. List of instructions**

Positive:

1. People will admire you
2. You will make good and lasting friendships
3. You will have lots of energy and enthusiasm
4. You will do well in you studies or work
5. You will achieve the things you set out to do
6. People you meet will like you
7. You will be very fit and healthy
8. You will be able to cope easily with pressure
9. You will have lots of good times with friends
10. Your mind will be very alert and “on the ball”

Negative:

1. You will have a serious disagreement with a good friend
2. You will have health problems
3. You will make a decision you regret
4. You will feel misunderstood
5. You will get the blame for things going wrong
6. You will be the victim of a crime
7. Someone close to you will reject you
8. People will dislike you
9. People will find you dull and boring
10. Things won’t work out as you had hoped

Neutral:

1. A bird flies by your window
2. You are having breakfast
3. Your phone rings
4. You are riding on a bus
5. You are talking to a colleague
6. You are in a library
7. You see someone walking a dog
8. You study/work just as usual
9. You see a car drive by
10. You are looking at the rain through a window
