# Supplementary material for: Physiological and subjective arousal to prospective mental imagery: A mechanism for behavioral change?
Source: PLoS One. 2023 Dec 12;18(12):e0294629. doi: 10.1371/journal.pone.0294629 (PMC10715665; doi:10.1371/journal.pone.0294629)
Supplement: S2 Table — The standard error of values are presented within parentheses. (PDF) [file pone.0294629.s002.pdf]

S2 Table. Descriptive statistics of primary analyses.
The standard error of values are presented within parentheses.

		Emotional Valence		
	N	Positive	Neutral	Negative
Skin conductance Response	53	0.79 (.06)	0.65 (.05)	0.75 (.07)
Vividness Ratings	60	3.26 (.09)	3.95 (.08)	2.97 (.09)
Arousal Ratings	60	43.68 (2.01)	29.68 (1.66)	45.34 (1.98)
Scene Construction Time	60	15.34 (0.69)	13.67 (0.74)	15.98 (0.64)
