# Supplementary material for: Physiological and subjective arousal to prospective mental imagery: A mechanism for behavioral change?
Source: PLoS One. 2023 Dec 12;18(12):e0294629. doi: 10.1371/journal.pone.0294629 (PMC10715665; doi:10.1371/journal.pone.0294629)
Supplement: S3 Table — (PDF) [file pone.0294629.s003.pdf]

S3 Table. ANOVA-table for emotional valence (positive, neutral, negative) with SCRs as the dependent variable (n=53).

	SS	*df*	*MS*	*F*	*P*	η_p^2
Emotional valence	0.542	2	0.271	2.795	0.066	0.05
Error	0.590	104	0.097			

Note. SCRs are root transformed and range corrected.
