# Supplementary material for: Physiological and subjective arousal to prospective mental imagery: A mechanism for behavioral change?
Source: PLoS One. 2023 Dec 12;18(12):e0294629. doi: 10.1371/journal.pone.0294629 (PMC10715665; doi:10.1371/journal.pone.0294629)
Supplement: S4 Table — (PDF) [file pone.0294629.s004.pdf]

S4 Table. Pairwise comparisons (positive, neutral, negative) with SCRs as the dependent variable (n=53).

	df	*t*	*P*	*d*
Positive vs. Neutral	52	2.42	0.02	0.33
Negative vs. Neutral	52	1.73	0.09	0.24
Positive vs. Negative	52	0.59	0.56	0.08

Note. SCRs are root transformed and range corrected.
