# Supplementary material for: Physiological and subjective arousal to prospective mental imagery: A mechanism for behavioral change?
Source: PLoS One. 2023 Dec 12;18(12):e0294629. doi: 10.1371/journal.pone.0294629 (PMC10715665; doi:10.1371/journal.pone.0294629)
Supplement: S5 Table — (PDF) [file pone.0294629.s005.pdf]

S5 Table. ANOVA-table for emotional valence (positive, neutral, negative) with vividness ratings as the dependent variable (n=60).

	SS	*df*	*MS*	*F*	*p*	η_p^2
Emotional valence	30.359	2	15.180	115.405	<0.001	0.66
Error	15.521	118	0.132			
