# Supplementary material for: Physiological and subjective arousal to prospective mental imagery: A mechanism for behavioral change?
Source: PLoS One. 2023 Dec 12;18(12):e0294629. doi: 10.1371/journal.pone.0294629 (PMC10715665; doi:10.1371/journal.pone.0294629)
Supplement: S6 Table — (PDF) [file pone.0294629.s006.pdf]

S6 Table. ANOVA-table for emotional valence (positive, neutral, negative) with arousal ratings as the dependent variable (n=60).

	SS	*df*	*MS*	*F*	*p*	η_p^2
Emotional valence	8877.847	1.774	5004.848	46.546	<0.001	0.44
Error	11253.227	104.657	107.525			

Note. Greenhouse-Geisser correction was used in this analysis.
