# Supplementary material for: Physiological and subjective arousal to prospective mental imagery: A mechanism for behavioral change?
Source: PLoS One. 2023 Dec 12;18(12):e0294629. doi: 10.1371/journal.pone.0294629 (PMC10715665; doi:10.1371/journal.pone.0294629)
Supplement: S7 Table — (PDF) [file pone.0294629.s007.pdf]

S7 Table. ANOVA-table for emotional valence (positive, neutral, negative) with scene construction time as the dependent variable (n=60).

	SS	*df*	*MS*	*F*	*p*	η_p^2
Emotional valence	1711521154.9	1.381	123890235.4	39.307	<0.001	0.40
Error	256900308.0	81.507	3151862.911			

Note. Greenhouse-Geisser correction was used in this analysis.
