# Supplementary material for: Physiological and subjective arousal to prospective mental imagery: A mechanism for behavioral change?
Source: PLoS One. 2023 Dec 12;18(12):e0294629. doi: 10.1371/journal.pone.0294629 (PMC10715665; doi:10.1371/journal.pone.0294629)
Supplement: S9 Table — (PDF) [file pone.0294629.s009.pdf]

S9Table. ANOVA table with emotional valence (positive, neutral, negative) and anxiety (high/low) with skin conductance as the dependent variable (N=53).

	SS	*df*	*MS*	*F*	*p*	η_p^2
Emotional valence	0.535	2	0.267	2.787	0.66	0.052
Emotional valence × Anxiety	0.297	2	0.149	1.548	0.22	0.029
Error (Emotional valence)	9.791	102	0.096			
Between-subjects effect						
Anxiety	1.186	1.000	1.186	3.344	0.07	0.062
Error	18.083	51	0.355			
