# Supplementary material for: Physiological and subjective arousal to prospective mental imagery: A mechanism for behavioral change?
Source: PLoS One. 2023 Dec 12;18(12):e0294629. doi: 10.1371/journal.pone.0294629 (PMC10715665; doi:10.1371/journal.pone.0294629)
Supplement: S10 Table — (PDF) [file pone.0294629.s010.pdf]

S10 Table. ANOVA table with emotional valence (positive, neutral, negative) and anxiety (high/low) with vividness ratings as the dependent variable (N=59).

	SS	*Df*	*MS*	*F*	*p*	η_p^2
Emotional valence	28.310	2	14.155	112.410	<0.001	0.66
Emotional valence × Anxiety	0.829	2	0.415	3.293	0.04	0.055
Error (Emotional valence)	14.355	114	0.126			
Between-subjects effect						
Anxiety	4.824	1.000	4.824	4.607	0.036	0.075
Error	59.677	57	1.047			
