# Supplementary material for: Physiological and subjective arousal to prospective mental imagery: A mechanism for behavioral change?
Source: PLoS One. 2023 Dec 12;18(12):e0294629. doi: 10.1371/journal.pone.0294629 (PMC10715665; doi:10.1371/journal.pone.0294629)
Supplement: S11 Table — (PDF) [file pone.0294629.s011.pdf]

S11 Table. ANOVA table with emotional valence (positive, neutral, negative) and anxiety (high/low) with arousal ratings as the dependent variable (N=59).

	SS	*df*	*MS*	*F*	*p*	η_p^2
Emotional valence	8417.875	1.736	4848.047	45.992	<0.001	0.45
Emotional valence × Anxiety	762.05	1.736	438.882	4.164	0.023	0.07
Error (Emotional valence)	10432	98.972	105.411			
Between-subjects effect						
Anxiety	209.585	1.000	209.585	0.463	0.499	0.008
Error	25788.116	57	452.423			

Note. Greenhouse-Geisser correction was used in this analysis.
