# Supplementary material for: Physiological and subjective arousal to prospective mental imagery: A mechanism for behavioral change?
Source: PLoS One. 2023 Dec 12;18(12):e0294629. doi: 10.1371/journal.pone.0294629 (PMC10715665; doi:10.1371/journal.pone.0294629)
Supplement: S12 Table — (PDF) [file pone.0294629.s012.pdf]

S12 Table. ANOVA table with emotional valence (positive, neutral, negative) and anxiety (high/low) with scene construction time as the dependent variable (N=59).

	SS	*df*	*MS*	*F*	*p*	η_p^2
Emotional valence	157115681.8	1.358	78587850.92	36.383	<0.001	0.39
Emotional valence × Anxiety	5028776.75	1.358	3703906.27	1.164	.30	0.02
Error (Emotional valence)	246242740.1	77.389	3181897.69			
Between-subjects effect						
Anxiety	279304247.4	1.000	279304247.4	3.501	0.066	0.58
Error	4547267616	57	79776624.83			

Note. Greenhouse-Geisser correction was used in this analysis.
