# Supplementary material for: Physiological and subjective arousal to prospective mental imagery: A mechanism for behavioral change?
Source: PLoS One. 2023 Dec 12;18(12):e0294629. doi: 10.1371/journal.pone.0294629 (PMC10715665; doi:10.1371/journal.pone.0294629)
Supplement: S14 Table — (PDF) [file pone.0294629.s014.pdf]

S14 Table. ANOVA table with emotional valence (positive, neutral, negative) and anxiety as a covariate, with skin conductance as the dependent variable (N=53).

	SS	*df*	*MS*	*F*	*p*	η_p^2
Emotional valence	0.284	2	0.142	1.475	0.234	0.028
Emotional valence × Anxiety	0.258	2	0.129	1.341	0.266	0.026
Error (Emotional valence)	9.830	102	0.096			
Between-subjects effect						
Anxiety	0.202	1	0.202	0.539	0.466	0.01
Error	19.067	51	0.374			
