# Supplementary material for: Physiological and subjective arousal to prospective mental imagery: A mechanism for behavioral change?
Source: PLoS One. 2023 Dec 12;18(12):e0294629. doi: 10.1371/journal.pone.0294629 (PMC10715665; doi:10.1371/journal.pone.0294629)
Supplement: S15 Table — (PDF) [file pone.0294629.s015.pdf]

S15 Table. ANOVA table with emotional valence (positive, neutral, negative) and anxiety as a covariate, with vividness ratings as the dependent variable (N=59).

	SS	*Df*	*MS*	*F*	*p*	η_p^2
Emotional valence	5.779	2	2.889	23.938	<0.001	0.296
Emotional valence × Anxiety	1.424	2	0.712	5.900	0.004	0.094
Error (Emotional valence)	13.760	114	0.121			
Between-subjects effect						
Anxiety	5.806	1	5.806	5.638	0.021	0.090
Error	58.695	57	1.030			
