# Supplementary material for: Physiological and subjective arousal to prospective mental imagery: A mechanism for behavioral change?
Source: PLoS One. 2023 Dec 12;18(12):e0294629. doi: 10.1371/journal.pone.0294629 (PMC10715665; doi:10.1371/journal.pone.0294629)
Supplement: S16 Table — (PDF) [file pone.0294629.s016.pdf]

S16 Table. ANOVA table with emotional valence (positive, neutral, negative) and anxiety as acovariate, with arousal ratings as the dependent variable (N=59).

	SS	*df*	*MS*	*F*	*p*	η_p^2
Emotional valence	2454.470	1.703	1441.495	13.461	<0.001	0.191
Emotional valence × Anxiety	801,100	1.703	470.481	4.393	0.02	0.072
Error (Emotional valence)	10393.602	97.055	107.089			
Between-subjects effect						
Anxiety	234.897	1	234.897	0.520	0.474	0.009
Error	25762.802	57	451.979			

Note. Greenhouse-Geisser correction was used in this analysis.
