# Supplementary material for: Physiological and subjective arousal to prospective mental imagery: A mechanism for behavioral change?
Source: PLoS One. 2023 Dec 12;18(12):e0294629. doi: 10.1371/journal.pone.0294629 (PMC10715665; doi:10.1371/journal.pone.0294629)
Supplement: S17 Table — (PDF) [file pone.0294629.s017.pdf]

S17 Table. ANOVA table with emotional valence (positive, neutral, negative) and anxiety as a covariate, with scene construction time as the dependent variable (N=59).

	SS	*df*	*MS*	*F*	*p*	η_p^2
Emotional valence	27125073.15	1.370	19792834.64	6.288	0.003	0.099
Emotional valence × Anxiety	5394039.035	1.370	3935964.415	1.250	0.29	0.021
Error (Emotional valence)	245877477.8	78.116	3147610.404			
Between-subjects effect						
Anxiety	503406453.4	1	503406453.4	6.637	0.013	0.104
Error	4323165409	57	75845007.18			

Note. Greenhouse-Geisser correction was used in this analysis.
