# Supplementary material for: Physiological and subjective arousal to prospective mental imagery: A mechanism for behavioral change?
Source: PLoS One. 2023 Dec 12;18(12):e0294629. doi: 10.1371/journal.pone.0294629 (PMC10715665; doi:10.1371/journal.pone.0294629)
Supplement: S18 Table — (PDF) [file pone.0294629.s018.pdf]

S18 Table. ANOVA table with emotional valence (positive, neutral, negative) and anxiety (high/low) with skin conductance as the dependent variable (N=53).

	SS	*df*	*MS*	*F*	*p*	η_p^2
Emotional valence	0.544	2	0.272	2.813	0.065	0.052
Emotional valence × Depression	0.221	2	0.110	1.141	0.323	0.022
Error (Emotional valence)	9.868	102	0.097			
Between-subjects effect						
Depression	1.077	1.000	1.077	3.021	0.088	0.056
Error	18.192	51	0.357			
