# Supplementary material for: Physiological and subjective arousal to prospective mental imagery: A mechanism for behavioral change?
Source: PLoS One. 2023 Dec 12;18(12):e0294629. doi: 10.1371/journal.pone.0294629 (PMC10715665; doi:10.1371/journal.pone.0294629)
Supplement: S19 Table — (PDF) [file pone.0294629.s019.pdf]

S19 Table. Comparisons between high and low symptoms of depression on positive, neutral, negative prospective imagery with SCRs as the dependent variable (n=53).

	df	*t*	*P*	*d*
Positive imagery	51	0.88	0.38	0.24
Neutral imagery	51	1.15	0.26	0.32
Negative imagery	51	2.09	0.04	0.58

Note. SCRs are root transformed and range corrected.
