# Supplementary material for: Physiological and subjective arousal to prospective mental imagery: A mechanism for behavioral change?
Source: PLoS One. 2023 Dec 12;18(12):e0294629. doi: 10.1371/journal.pone.0294629 (PMC10715665; doi:10.1371/journal.pone.0294629)
Supplement: S20 Table — (PDF) [file pone.0294629.s020.pdf]

S20 Table. ANOVA table with emotional valence (positive, neutral, negative) and anxiety (high/low) with vividness ratings as the dependent variable (N=59).

	SS	*df*	*MS*	*F*	*p*	η_p^2
Emotional valence	28.619	2	14.310	116.698	<0.001	0.67
Emotional valence × Depression	1.206	2	0.603	4.916	0.009	0.079
Error (Emotional valence)	13.979	114	0.123			
Between-subjects effect						
Depression	0.214	1.000	0.214	0.190	0.665	0.003
Error	64.287	57	1.128			
