# Supplementary material for: Physiological and subjective arousal to prospective mental imagery: A mechanism for behavioral change?
Source: PLoS One. 2023 Dec 12;18(12):e0294629. doi: 10.1371/journal.pone.0294629 (PMC10715665; doi:10.1371/journal.pone.0294629)
Supplement: S21 Table — (PDF) [file pone.0294629.s021.pdf]

S21 Table. ANOVA table with emotional valence (positive, neutral, negative) and anxiety (high/low) with arousal ratings as the dependent variable (N=59).

	SS	*df*	*MS*	*F*	*p*	η_p^2
Emotional valence	8642.082	1.815	4761.140	47.393	<0.001	0.45
Emotional valence × Depression	800.764	1.815	441.161	4.391	0.018	0.072
Error (Emotional valence)	10393.94	103.462	100.461			
Between-subjects effect						
Depression	1485.882	1.000	1485.882	3.455	0.068	0.057
Error	24511.82	57	430.032			

Note. Greenhouse-Geisser correction was used in this analysis.
