# Supplementary material for: Physiological and subjective arousal to prospective mental imagery: A mechanism for behavioral change?
Source: PLoS One. 2023 Dec 12;18(12):e0294629. doi: 10.1371/journal.pone.0294629 (PMC10715665; doi:10.1371/journal.pone.0294629)
Supplement: S22 Table — (PDF) [file pone.0294629.s022.pdf]

S22 Table. ANOVA table with emotional valence (positive, neutral, negative) and anxiety (high/low) with scene construction time as the dependent variable (N=59).

	SS	*df*	*MS*	*F*	*p*	η_p^2
Emotional valence	157906359.5	1.387	78953179.76	37.087	<0.001	0.39
Emotional valence × Depression	8582457.03	1.387	6187691.72	2.016	0.153	0.034
Error (Emotional valence)	242689059.8	79.06	3069674.839			
Between-subjects effect						
Depression	329466745.6	1.000	329466745.6	4.176	0.046	0.068
Error	4497105117	57	78896581			

Note. Greenhouse-Geisser correction was used in this analysis.
