# Supplementary material for: Physiological and subjective arousal to prospective mental imagery: A mechanism for behavioral change?
Source: PLoS One. 2023 Dec 12;18(12):e0294629. doi: 10.1371/journal.pone.0294629 (PMC10715665; doi:10.1371/journal.pone.0294629)
Supplement: S23 Table — (PDF) [file pone.0294629.s023.pdf]

S23 Table. Comparisons between high and low symptoms of depression on positive, neutral, negative prospective imagery with Scene construction time as the dependent variable (N=59).

	df	*t*	*P*	*d*
Positive imagery	57	-1.87	0.07	-0.49
Neutral imagery	57	-2.29	0.03	-0.60
Negative imagery	57	-1.79	0.08	-0.47
