# Supplementary material for: Physiological and subjective arousal to prospective mental imagery: A mechanism for behavioral change?
Source: PLoS One. 2023 Dec 12;18(12):e0294629. doi: 10.1371/journal.pone.0294629 (PMC10715665; doi:10.1371/journal.pone.0294629)
Supplement: S24 Table — (PDF) [file pone.0294629.s024.pdf]

S24 Table. ANOVA table with emotional valence (positive, neutral, negative) and depression as a covariate, with skin conductance as the dependent variable (N=53).

	SS	*df*	*MS*	*F*	*p*	η_p^2
Emotional valence	0.248	2	0.124	1.268	0.286	0.024
Emotional valence × Depression	0.107	2	0.054	0.549	0.579	0.011
Error (Emotional valence)	9.981	102	0.098			
Between-subjects effect						
Depression	0.210	1	0.210	0.562	0.457	0.011
Error	19.059	51	0.347			
