# Supplementary material for: Physiological and subjective arousal to prospective mental imagery: A mechanism for behavioral change?
Source: PLoS One. 2023 Dec 12;18(12):e0294629. doi: 10.1371/journal.pone.0294629 (PMC10715665; doi:10.1371/journal.pone.0294629)
Supplement: S25 Table — (PDF) [file pone.0294629.s025.pdf]

S25 Table. ANOVA table with emotional valence (positive, neutral, negative) and depression as a covariate, with vividness ratings as the dependent variable (N=59).

	SS	*df*	*MS*	*F*	*p*	η_p^2
Emotional valence	18.657	2	9.328	77.330	<0.001	0.576
Emotional valence × Depression	1.432	2	0.716	5.937	0.004	0.094
Error (Emotional valence)	13.752	114	0.121			
Between-subjects effect						
Depression	1.260	1	1.260	1.136	0.291	0.020
Error	63.241	57	1.109			
