# Supplementary material for: Physiological and subjective arousal to prospective mental imagery: A mechanism for behavioral change?
Source: PLoS One. 2023 Dec 12;18(12):e0294629. doi: 10.1371/journal.pone.0294629 (PMC10715665; doi:10.1371/journal.pone.0294629)
Supplement: S26 Table — (PDF) [file pone.0294629.s026.pdf]

S26 Table. ANOVA table with emotional valence (positive, neutral, negative) and depression as a covariate, with arousal ratings as the dependent variable (N=59).

	SS	*df*	*MS*	*F*	*p*	η_p^2
Emotional valence	6098.159	1.769	3049.079	32.713	<0.001	0.365
Emotional valence × Depression	569.251	1.769	321.838	3.054	0.058	0.051
Error (Emotional valence)	10625.45	100.819	105.392			
Between-subjects effect						
Depression	164.066	1	164.066	0.362	0.55	0.006
Error	25833.64	57	453.222			

Note. Greenhouse-Geisser correction was used in this analysis.
