# Supplementary material for: Physiological and subjective arousal to prospective mental imagery: A mechanism for behavioral change?
Source: PLoS One. 2023 Dec 12;18(12):e0294629. doi: 10.1371/journal.pone.0294629 (PMC10715665; doi:10.1371/journal.pone.0294629)
Supplement: S27 Table — (PDF) [file pone.0294629.s027.pdf]

S27 Table. ANOVA table with emotional valence (positive, neutral, negative) and depression as a covariate, with scene construction time as the dependent variable (N=59).

	SS	*df*	*MS*	*F*	*p*	η_p^2
Emotional valence	89833089.80	1.373	65432221.90	20.692	<0.001	0.266
Emotional valence × Depression	3809873.439	1.373	2775018,473	0.878	0.384	0.015
Error (Emotional valence)	247461643.4	78.256	3162192.170			
Between-subjects effect						
Depression	278139932.1	1	278139932.1	3.486	0.067	0.058
Error	4548431931	57	79797051.42			

Note. Greenhouse-Geisser correction was used in this analysis.
